# Critical Insights Into Neonatal Hypertension: A Comprehensive Review of Current Understanding and Management Strategies

**DOI:** 10.7759/cureus.62421

**Published:** 2024-06-15

**Authors:** Amar Taksande, Rahul Khandelwal, Chaitanya Kumar Javvaji

**Affiliations:** 1 Pediatrics, Jawaharlal Nehru Medical College, Datta Meghe Institute of Higher Education and Research, Wardha, IND

**Keywords:** antihypertensive, oscillometric, neonates, blood pressure, hypertension

## Abstract

Hemodynamic adaptation to the extrauterine environment results in rapid blood pressure (BP) fluctuations during the neonatal period, particularly in premature infants. BP levels in neonates are influenced by several factors, including gestational age, birth weight, and maternal health. Diagnosing neonatal hypertension (HTN) typically requires a thorough diagnostic evaluation. Common etiologies include renal parenchymal disease, umbilical catheter-related thrombosis, and chronic lung disease. Despite the prevalence of HTN-associated factors and risk factors in neonates, management can be challenging. Fortunately, most cases of neonatal HTN resolve over time. This review explores these concepts and highlights the evidentiary gaps that need to be addressed.

## Introduction and background

The existence of neonatal hypertension (HTN) has long been acknowledged. Because gestational age and maturation impact blood pressure (BP) levels, it can be difficult to set standard BP values for newborns, particularly in preterm newborns. After delivery, BP levels rise; preterm newborns experience higher rates of rise than term infants [1]. Because there is little high-quality normative data, it is challenging to define HTN in neonates. However, it is evident that certain neonates have HTN, which can be severe and lead to major morbidity [1-3]. Neonates that have HTN often have thromboembolic events associated with umbilical catheterization, congenital issues such as aortic coarctation, renovascular disease, structural renal abnormalities, acquired renal illness, and certain drugs. In most situations, the probable reason can be ascertained with a thorough history and physical examination, negating costly laboratory or radiologic tests. It is becoming clear that managing a newborn's HTN is crucial not just in the acute or critical care context but also long after the neonatal stage, as hypertensive neonates need constant monitoring.

HTN may be diagnosed in a baby if the systolic BP (SBP) and/or diastolic BP (DBP) values regularly exceed the 95th percentile for postconceptional age. Depending on the clinical context, neonatal HTN has different incidences. Around 1.5% to 2.5% of all neonates admitted to neonatal intensive care units (NICUs) have it [4-5]. HTN is extremely rare in otherwise healthy newborns born at term gestation, with a reported prevalence of 0.2% [5-6]. The use of umbilical arterial catheters, acute kidney injury (AKI), heart disease, bronchopulmonary dysplasia (BPD), premature infants and infants with birth weight less than 2.5 kg (low birth weight (LBW)), and the severity of the illness increasing are some of the risk factors linked to neonatal HTN in newborns admitted to NICU. A study by Sahu et al. [7] identified the main risk factors for preterm infants as BPD and iatrogenic factors.

Conversely, systemic diseases were found to be the primary risk factors for term infants. Perinatal risk factors cause a substantial portion of neonatal HTN. A retrospective study conducted by Seliem et al. [8] involving neonates diagnosed with systemic HTN reported that infants with HTN exhibited significant LBW and short length. Lower gestational age on the other hand, umbilical arterial catheter use, patent ductus arteriosus, antenatal steroid administration, postnatal acute renal failure, maternal HTN and indomethacin treatment were associated with a higher likelihood of neonatal HTN (odds ratios (ORs) of 10.0, 5.9, 8.7, 51.8, 3.8, and 5.7, respectively). SBP or DBP that consistently surpasses the 95th percentile for postmenstrual age (PMA) is referred to as neonatal HTN. Monitoring and investigation are necessary for patients exceeding the 95th and 99th percentiles, respectively [9-10]. The baby's normal BP readings depend on gender, postnatal age, and measurement method (invasive vs noninvasive). Preterm levels are lower and change with gestation. If SBP and/or DBP measurements are at or above the postconceptual age 95th percentile on three different occasions, HTN is diagnosed [11-13]. Dionne et al. [14] compiled available BP measurements for neonates over two weeks old from existing literature and formulated the Table 1 to include the 50th, 95th, and 99th percentiles for mean arterial pressure (MAP), SBP, and DBP.

**Table 1:** Neonatal blood pressures by postmenstrual age after two weeks of life SBP: Systolic blood pressure; DBP: diastolic blood pressure; MAP: mean arterial pressure

Postmenstrual age	50th percentile	95th percentile	99th percentile
SBP 44 weeks	88	105	110
DBP 44 weeks	50	68	73
MAP 44 weeks	63	80	85
SBP 42 weeks	85	98	102
DBP 42 weeks	50	65	70
MAP 42 weeks	62	76	81
SBP 40 weeks	80	95	100
DBP 40 weeks	50	65	70
MAP 40 weeks	60	75	80
SBP 38 weeks	77	92	97
DBP 38 weeks	50	65	70
MAP 38 weeks	59	74	79
SBP 36 weeks	72	87	92
DBP 36 weeks	50	65	70
MAP 36 weeks	57	72	77
SBP 34 weeks	70	85	90
DBP 34 weeks	40	55	60
MAP 34 weeks	50	65	70
SBP 32 weeks	68	83	88
DBP 32 weeks	40	55	60
MAP 32 weeks	49	64	69
SBP 30 weeks	65	80	85
DBP 30 weeks	40	55	60
MAP 30 weeks	48	63	68
SBP 28 weeks	60	75	80
DBP 28 weeks	38	50	54
MAP 28 weeks	45	58	63

## Review

The most common causes of neonatal HTN, even though several have been found, are renal disease, chronic lung illness, and umbilical catheter-associated thromboembolism. Sometimes, though, an explicit underlying cause remains elusive. Undetectable renovascular events may be the cause of HTN in people for whom there is no known explanation (Table 2).

**Table 2:** Causes of neonatal hypertension PDA: Patent ductus arteriosus; TPN: total parenteral nutrition

Renovascular causes	Renal artery thrombosis, thromboembolism, renal artery stenosis, renal venous thrombosis, renal artery compression
Renal parenchymal disease	Congenital nephrotic syndrome, glomerulonephritis, pyelonephritis, Wilms tumor
Acquired renal conditions	Acute tubular necrosis, cortical necrosis, interstitial nephritis, hemolytic uremic syndrome (HUS)
Congenital disorders	Obstructive uropathy, polycystic kidney disease, multicystic dysplastic kidney disease, unilateral renal hypoplasia
Endocrine	Congenital adrenal hyperplasia (CAH), primary hyperaldosteronism, hyperthyroidism
Cardiovascular system	Coarctation of aorta, Post-PDA ligation, ductal aneurysm
Neurological	Cushing's disease, neural crest tumor, cerebral angiomas, interventricular hemorrhage (IVH) subdural hematoma, neonatal seizures
Pulmonary	Chronic lung disease (CLD), bronchopulmonary dysplasia (BPD), pneumothorax
Medications	Neonatal drugs: steroids-dexamethasone; inotropic-dopamine, dobutamine, adrenaline toxicity of vitamin D, methyl xanthine/theophylline, caffeine, pancuronium, phenylephrine. Maternal drugs: cocaine, heroin
Miscellaneous	Birth asphyxia, TPN, closure of abdominal wall defect like omphalocele, gastroschisis, adrenal hemorrhage, adrenal tumors, hypercalcemia

The most common pathway, active in most diseases resulting in neonatal HTN, is renovascular. Numerous different systemic factors can cause reduced renal perfusion. Blood clots occur in the renal arteries, and endothelial cells are damaged due to umbilical artery catheterization. Consequently, there is a decreased kidney perfusion, which sets off the renin-angiotensin-aldosterone system (RAAS) and results in HTN [15]. It has been shown that BPD is associated with elevated serum aldosterone levels and an incapacity to eliminate free water, both of which may exacerbate systemic HTN. Neonatal HTN in babies with BPD is considered etiopathogenesis by aberrant vasomotor functions and an increase in peripheral artery wall thickness, according to recent research [16-17]. Reduced renal perfusion and renal vein thrombosis (RVT) can be observed in infants delivered to mothers with diabetes or who carry the Factor V Leiden gene. Renal tubular necrosis (ATN) can result from neonatal asphyxia, which can then cause neonatal HTN. By compression of the renal arteries or ureters or by releasing vasoactive chemicals, newborn tumors can result in HTN. Examples of these tumors include pheochromocytoma, neuroblastoma, Wilms tumor, and mesoblastic nephroma. RAAS activation decreased renal perfusion and reduced forward blood flow are the outcomes of coarctation of the aorta (COA) and patent ductusarteriosus (PDA). Renal vascular compression may result from increased intraabdominal pressure brought on by the closure of abdominal wall defects. The causes of HTN in extracorporeal membrane oxygenation (ECMO) patients are believed to be an increase in stroke volume due to the increase in aortic return from the ECMO pump blood, as well as improper handling of NA or water in regard to the infants' arterial flow through their systems. The study by Goetzman et al. [18] revealed that out of 98 newborn infants who underwent umbilical artery catheterization, 23 (24%) experienced a thrombotic complication as determined by aortography. No relationship was seen between the duration of time the catheters were inserted into the umbilical arteries and the incidence of thrombotic problems. Twelve infants with HTN were studied by Adelman [19]. Ten neonates received umbilical artery catheters, and nine positioned above the renal artery origin. Eleven newborns had renal artery thrombosis, the most common kind of renovascular illness, according to radionuclide renal scan and/or angiography results. According to Abman et al. [20], SBP values exceeding 113 mm of Hg were observed at least three distinct times in 13 out of 30 babies diagnosed with BPD. By comparison, just one baby out of 22 that did not have BPD experienced HTN. The author advocated careful BP monitoring throughout the infant's follow-up treatment, concluding that systemic HTN is a severe problem for newborns with BPD.

Phthalate exposure

Phthalates, including di-2-ethylhezyl phthalate (DEHP), is a plasticizing chemical that is usually found in a continuous positive airway pressure device, certain endotracheal tubes, and intravenous (IV) bags and tubing. One study found a possible connection between phthalate exposure and "idiopathic" neonatal HTN, which usually appears 40 weeks after PMA, is transitory, has low plasma renin activity, and responds well to spironolactone [21]. According to one study, 11-beta hydroxysteroid dehydrogenase 2 suppression activates the mineralocorticoid receptor. A study conducted at the same facility found that the incidence of newborn HTN dropped to 7.7% when DEHP-containing IV fluids were stopped, but it increased to 10.1% when they were resumed [22]. These observations have not yet been independently confirmed.

Measurement of BP in neonates

BP measurement in newborns, especially preterm infants, is challenging. The gold standard is invasive intra-arterial measurement, typically performed in a NICU using an umbilical artery catheter, though noninvasive oscillometric devices are now universally used due to their simplicity and ability to frequently measure BP without causing harm. These devices measure MAP, with calculations of systolic and diastolic pressures . Since crying, pain, nursing, and agitation can all raise BP, it is best to take the baby's blood pressure while they are sleeping or relaxing [23].

Intra-arterial Measurement

**Figure 1:**
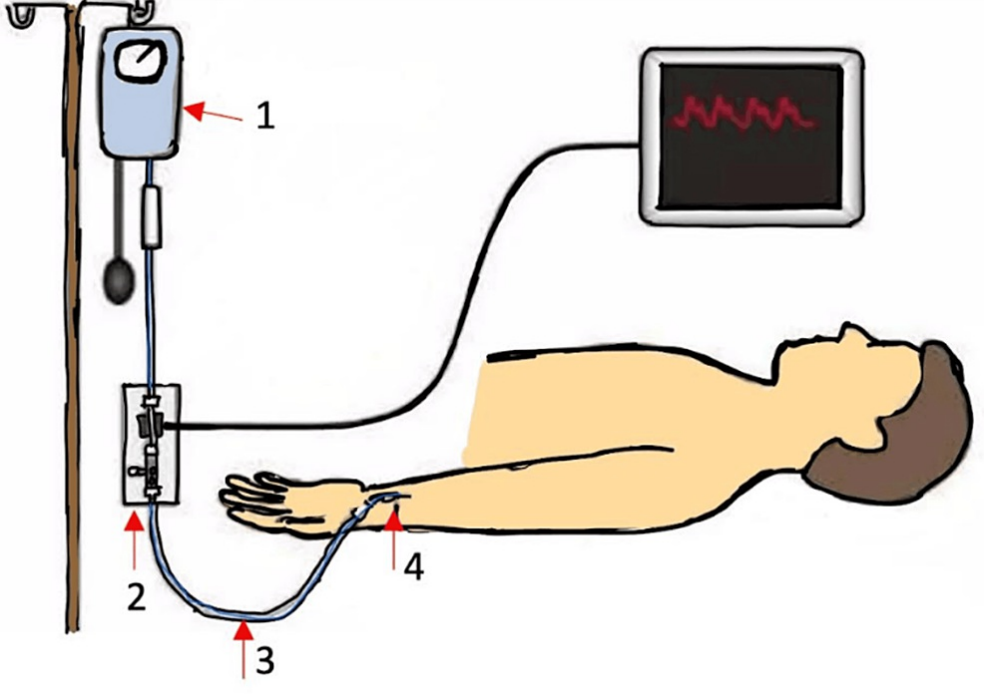
Intra-arterial measurement: the most accurate technique for measuring blood pressure (1) Pressure bag; (2) pressure and transducer and automatic flushing system; (3) saline-filled noncompressible tubing; (4) arterial line

The most reliable way to directly measure BP is by inserting a catheter into the aorta or radial artery, as shown in Figure 1. This method allows for continuous monitoring of BP. However, there is some uncertainty regarding the correlation between pressures in the radial artery and the aorta. In adults, SBP readings from the radial artery can be 20-30% higher than those from central arteries, although mean and diastolic pressures tend to be similar. In newborns, radial pressures seem to be more similar to aortic pressures [24]. However, using intra-arterial catheters comes with risks like thrombosis and infection. Because of these complications, such catheters should only be used for monitoring BP when there's a strong clinical need, such as frequent blood tests, hypotension needing pressor agents, or severe HTN requiring IV medications [25].

Noninvasive Measurement

**Figure 2:**
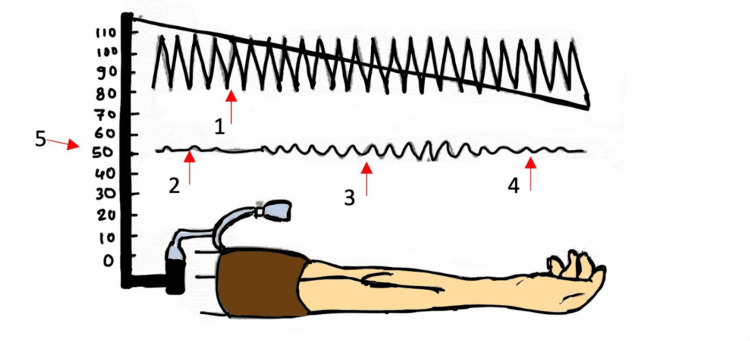
Noninvasive measurement: the most common technique is oscillometry (1) Cuff pressure oscillations; (2) systolic blood pressure; (3) mean arterial pressure; (4) diastolic blood pressure; (5) pressure in mmHg

There are many noninvasive devices for measuring BP, with oscillometry being the most common method. This technique estimates the average arterial pressure and then calculates the SBP and DBP from that value, as shown in Figure 2. However, the algorithms used for these calculations differ among manufacturers, which can lead to varying BP readings for the same patient depending on the device used. A review of the literature shows that the accuracy of oscillometric BP measurements relies on correct techniques. This involves using a properly sized cuff, with a width to arm circumference ratio of 0.45-0.55 and always measuring BP on the upper arm. Although these devices are usually considered reliable compared to intra-arterial measurements, some studies have found notable differences. A systematic literature review has found that oscillometric BP measurements are most accurate for MAP when compared to intra-arterial measurements. However, SBP, DBP, and MAP measured using oscillometry are generally less accurate and precise than those obtained via intra-arterial methods, particularly in neonates with a MAP below 30 mmHg. Although these discoveries have been made, oscillometric devices remain valuable for measuring BP in the NICU due to their ability to provide ongoing monitoring and track BP trends. Nevertheless, healthcare providers must keep in mind that oscillometric BP readings might give higher values than actual intra-arterial BP.

Cuff Size

Using the proper size cuff is crucial for noninvasive BP monitoring. The optimal length of the cuff bladder should be two thirds the length of the extremities, while the ideal ratio between the cuff width and arm circumference should fall within the range of 0.44 to 0.55 [10]. Opting for a larger cuff would be a better choice in case one has to decide between a cuff that is too small or too large, as it would minimize errors. The majority of normal BP readings are obtained from the upper arm, making it the preferred location for BP monitoring [26-28]. However, if the lower arm is being measured, a cuff that is two thirds the length of the arm should be selected.

Clinical features

Most newborns with HTN do not show any symptoms, and the diagnosis is made through routine measurement of BP. When symptoms or signs are present, the severity of HTN may not necessarily correspond to its presence or intensity. HTN can be linked to various symptoms and vague symptoms, such as apnea, lethargy, and poor feeding, as well as cardiorespiratory, neurological, and renal symptoms (Table 3).

**Table 3:** Associated signs and symptoms of neonatal hypertension AKI: Acute kidney injury

Cardiorespiratory signs	Tachypnea, tachycardia, cyanosis, cardiomegaly, mottling, heart failure, cardiogenic shock
Neurological symptoms	Irritability, lethargy, hypotonia, hypertonia, seizures, hemiparesis, hypertensive encephalopathy, hypertensive retinopathy
Renal abnormalities	Oliguria, polyuria, hematuria, sodium wasting, AKI

Neonatal HTN can lead to life-threatening complications, including cardiogenic shock, cerebral hemorrhage, neonatal seizures, congestive cardiac failure, and hypertensive retinopathy [25]. In some instances, the clinical manifestations associated with HTN can also be attributed to the underlying cause [28-29]. Investigations should be conducted if persistent SBP and/or DBP readings are above the 95th percentile for PMA. Confirming the diagnosis of HTN through frequent, precise measurements is the initial step in the evaluation process [30-31]. Three or more increased BP readings over a 6- to 12-hour period are typically sufficient to support an HTN diagnosis. 

Evaluation of neonatal HTN

Neonatal HTN typically has an underlying cause and is rarely without a known cause. Therefore, it is essential to thoroughly evaluate and investigate each neonatal HTN in order to identify the underlying etiology. This evaluation involves a focused analysis of the patient's medical history, followed by a physical examination and imaging studies with laboratory tests. A specific history examines relevant prenatal exposures, the neonatal course, and other medical issues. Essential factors to consider in the perinatal history are prenatal exposures (e.g., medications, maternal diabetes), prenatal imaging results (e.g., renal vascular malformations, renal structural malformations, mass in the abdomen, and other malformations), and immediate postnatal complications (e.g., decreased blood pressure, meconium aspiration). An umbilical arterial catheter (UAC), medications (e.g., postnatal corticosteroids, vasopressors, caffeine, and theophylline), and the existence of concurrent conditions linked to HTN (e.g., congenital anomalies, BPD, and/or syndromes associated with HTN) are all examined in the neonatal history [9]. At least one BP measurement in each of the four extremities should be part of the physical examination to rule out aortic coarctation. A review of the BP measurement procedure and the correct positioning of the BP cuff is crucial. To determine a precise diagnosis, it is important to evaluate the infant's overall appearance for any dysmorphic traits. Any syndromic facies that can point to the existence of neonatal HTN should be looked for in the newborn. A disorder like Williams syndrome, which has HTN as one of its clinical symptoms, may be indicated by dysmorphic characteristics. Examining the cardiovascular system should include assessing for unusual cardiac sounds, palpating the pulses in the lower and upper limbs, measuring the heart rate, and evaluating for signs of heart failure. Tachycardia, poor perfusion, and tachypnea, as indicated by low systemic BP, decreased peripheral pulses, capillary refill, and cool and mottled extremities, may indicate heart failure. The abdomen should be examined to assess for the presence of mass (such as RVT, tumors, cystic kidney disease, polycystic kidney disease (PCKD)), while the genitourinary examination to check for any anomalies or virilization in cases of congenital adrenal hyperplasia. A neurologic examination is also necessary to look for signs of CNS dysfunction which are consistent with HTN encephalopathy and require immediate treatment [9]. In addition to the history and physical examination, further laboratory investigations will likely be needed.

Investigation

The initial investigations entail evaluating the renal function by assessing levels of blood urea nitrogen (BUN) and serum creatinine. A urine examination is conducted to detect the presence of hematuria, renal cast, or urinary tract infection (UTI), and a urine culture is performed to identify UTIs. The urine albumin/creatinine ratio and urine protein/creatinine ratio are also measured. To assess for certain conditions such as pseudohypoaldosteronism type II or congenital adrenal hyperplasia, serum electrolytes are analyzed, specifically hypokalemia or hyperkalemia. When renal vein thrombosis is present, thrombocytopenia is determined by doing a complete blood count. Measurements of calcium levels and arterial blood gas analysis are done to rule out hypercalcemia [9,32-33]. Radiography of the abdomen and an ultrasound examination of the suprarenal and renal regions are necessary for all hypertensive infants. To detect any thrombi, newborns with an indwelling umbilical artery catheter should have imaging of their renal and aortic arteries, ideally by ultrasonography. Renal vein thromboses, obstructive uropathies, tumors, parenchymal renal illness, and congenital anomalies of the renal tract can also be detected by these imaging modalities. Doppler studies of the renal arteries may indicate the presence of stenosis, although branch artery stenosis might not be detected. In addition, an echocardiography is carried out to assess heart function, specifically left ventricular dysfunction or decreased contractility, and look into possible cardiac causes. The brain is also scanned to detect intracranial hemorrhage and cerebral edema (Table 4).

**Table 4:** Investigations required in neonatal hypertension CBC: Complete blood count; KFT: kidney function tests; USG: ultrasonogram; PMA: postmenstrual age; CT: computed  tomography; MRI: magnetic resonance imaging

Routine investigations	CBC, KFT, urine analysis, urine culture, serum calcium levels, thyroid function test, serum aldosterone, coagulation profile
Imaging studies	Kidney USG with doppler evaluation, renal scintigraphy (PMA of 44 weeks) angiography-noninvasive renal angiography including CT scan, MRI, echocardiography neurosonography

A thorough study must be carried out if the preliminary analysis points to renal pathophysiology, which requires more assessment. This might include a dimercapto succinic acid (DMSA) scan to rule out arterial infarction, radionuclide scintigraphy (using diethylenetriaminepentaacetic acid (DTPA) or mercaptuacetyltriglycine (MAG3)) to reveal perfusion abnormalities, and avoiding cystoureterogram in situations of suspected urinary tract deformity. The purpose of these tests is to determine newborn HTN's underlying etiology. Percutaneous femoral renal arteriography and simultaneous assessment of bilateral renin level evaluated from the renal vein should be carried out if renal artery stenosis or thrombosis is considered the etiology of newborn HTN. The plasma renin activity is normal under these circumstances. To diagnose renovascular HTN, MRI is considered the gold standard. While renovascular disease is linked to increased plasma renin activity, primary hyperaldosteronism is related with lower plasma renin activity. Suppose a newborn is suspected of having an endocrine cause based on clinical examination. In that case, additional tests should be carried out, such as urinary 17-hydroxysteroids, serum cortisol, thyroid function tests, serum aldosterone and 17-ketosteroids for Cushing's syndrome and congenital adrenal hyperplasia, vanillylmandelic acid (VMA) for pheochromocytoma and urinary metanephrines for congenital neuroblastoma, and a urine toxicology screen in cases of suspected maternal drug addiction [34].

Management of neonatal HTN

In case of mild asymptomatic HTN (systolic BP: 95th to <99th percentile) in neonates may be observed closely. Drug treatment can be considered, if BP elevation is persistent (Table 5). In mild HTN with evidence of end-organ involvement, neonates with evidence of target organ involvement, mainly left ventricular hypertrophy detected by echocardiography should be started on antihypertensive therapy. In asymptomatic moderate HTN (BP ≥99th percentile), antihypertensive therapy should be started in neonates.

**Table 5:** Drugs used in neonatal hypertension OD: Omnie die (daily once ); BD: bis-in die (twice daily); TDS: ter in die (three times a day); QID: quarter in die (four times a day); ACE inhibitor: angiotensin-converting enzyme inhibitor; IV: intravenous

Class	Drug	Route	Dose range	Interval	Comments
Calcium channel blockers	Amlodipine	Oral	0.05 mg/kg/dose Max: 0.6 mg/kg/day	OD	May cause edema, tachycardia, and gingival hypertrophy
	Isradipine	Oral	0.05 to 0.15 mg/kg/dose Max: 0.8 mg/kg/day	QID	May cause hypotension, edema, and tachycardia
	Nicardipine	IV infusion	0.5 to 4 mcg/kg/minute	Continuous infusion	May cause hypotension, tachycardia, and flushing. Caution in perinatal asphyxia
Vasodilators	Hydralazine	Oral	0.25 to 1 mg/kg/dose Max: 5 mg/kg/day	TDS or QID	May cause fluid retention, tachycardia, diarrhea, emesis, and rare agranulocytosis
		IV bolus	0.15 to 0.6 mg/kg/dose	Q 4 hours	
	Sodium nitroprusside	IV infusion	Initial: 0.25 μg/kg/min Max: 8 μg/kg/min	Continuous infusion	May cause hypotension, tachycardia. Monitor for cyanide toxicity. Use caution in renal and hepatic failure
Diuretics	Chlorothiazide	Oral	5 to 15 mg/kg/dose	BD	
	Hydrochlorothiazide	Oral	1 to 3 mg/kg/day	OD or BD	May cause hyponatremia, hypokalemia, and alkalosis
	Spironolactone	Oral	0.5 to 1.5 mg/kg/dose	BD	May cause hyperkalemia. Caution in renal failure
Alpha and beta antagonists	Labetalol	Oral	0.5 to 1 mg/kg/dose Max: 10 mg/kg/day	BD or TDS	
		IV bolus	0.2 to 1 mg/kg/dose	Administer every 4 to 6 hours	
		IV infusion	0.25 to 3 mg/kg/hour	Continuous infusion	May cause hypotension, bradycardia, hyperkalemia, hypoglycemia, hyperglycemia, edema. Caution in chronic lung disease, heart block, and unstable heart failure
Beta antagonists	Esmolol	IV infusion	100 to 500 mcg/kg/minute	Continuous infusion	
	Propranolol	Oral	0.5 to 1 mg/kg/dose Max: 8 to 10 mg/kg/day	TDS or QID	May cause hypotension, bradycardia, hyperkalemia, hypoglycemia, hyperglycemia, edema. Use caution in chronic lung disease, heart block, and unstable heart failure
ACE inhibitors	Captopril	Oral	<3 months: 0.01 to 0.5 mg/kg/dose Max: 2 mg/kg/day	TDS or QID	
	Enalapril	Oral	0.08 to 0.6 mg/kg/day	OD or BD	

In severe symptomatic HTN, continuous infusions of IV antihypertensive medications should be used. Potent oral drugs and IV bolus infusions should be avoided as they have the potential to reduce BP too quickly and for a considerable amount of time. The treatment of HTN should follow a stepwise approach, beginning with reducing any treatments that may increase BP. Correction of salt or fluid overload is imperative. When deciding whether to proceed with surgery after investigations show that the lesion has a surgically curable etiology, factors such as the neonate's age and weight, the severity of HTN, and the advantages and disadvantages of medical therapy are considered. When seeking medical attention, one can choose from five types of antihypertensive medications angiotensin-converting enzyme (ACE) inhibitors or calcium channel blockers (CCBs) [33-35]. CCBs are typically prescribed orally as first-line treatments for HTN without a clear etiology. Since isradipine has a short half-life and a low risk of adverse effects, it is typically used in acute situations. Amlodipine acts more slowly initially and is usually started in a less severe or longer-term situation. Additional oral pharmaceutical choices include diuretics like thiazides, spironolactone, or vasodilators like hydralazine. Although they can be utilized, loop diuretics carry a greater risk of developing hypercalciuria, aggravating acute renal damage, and electrolyte imbalances. While beta-blockers like labetalol and propranolol can be used to treat neonatal HTN of chronic origin, especially when it comes to cardiac origin, they are generally avoided in newborns with chronic lung disease.

CCBs

CCBs, including nicardipine and isradipine, exhibit strong peripheral vasodilation properties and have shown efficacy in managing neonatal HTN. Nicardipine administered intravenously is commonly preferred as the primary treatment for severe HTN. Both oral and IV forms of CCB are accessible for administration. Isradipine has demonstrated efficacy as an oral CCB and has shown effectiveness in treating both acute and chronic HTN [35]. The therapeutic response to isradipine has been observed in both acute and chronic HTN cases, indicating its effectiveness [35]. Its action begins within one to two hours after ingestion, making it a suitable option for infants as it can be easily administered in a stable 1 mg/mL solution. Amlodipine, a long-acting CCB, is commonly utilized in managing HTN in children [36]. The utilization of short-acting nifedipine is discouraged due to its inability to be formulated into a stable oral form and its potential to induce sudden, drastic decreases in BP [37]. Nicardipine has demonstrated efficacy and safety in both term and preterm newborns with HTN stemming from various causes [38-39]. IV administration of nicardipine is widely regarded as the preferred treatment for severe HTN, as it rapidly reduces BP within minutes. Its short half-life of 10 to 15 minutes necessitates continuous infusion for optimal therapeutic effect. Nicardipine is initiated as a continuous IV infusion at a dosage of 0.5 mcg/kg per minute. At intervals of 15 minutes, the infusion may be escalated by 0.25 to 0.5 mcg/kg per minute, with a maximum limit of 3 mcg/kg per minute, if the desired BP goal is not achieved within that specific duration. In the exceptional case of a patient whose BP does not show improvement with the administration of nicardipine or whose BP remains high even after receiving the maximum dosage of nicardipine, it is possible to introduce or replace the treatment with infusions of either sodium nitroprusside or esmolol. Once the desired BP level is achieved, and if the patient's clinical condition allows, a switch to oral medication can be considered. It is worth noting that nicardipine has been proven to be safe and effective, with no significant adverse effects on the average heart rate in children following coarctectomy.

Vasodilators

Hydralazine, a direct vasodilator, induces relaxation of vascular smooth muscle leading to a reduction in BP. Its mechanism of action remains unclear. Both oral and IV administration of hydralazine have shown potential in managing moderate HTN in infants. Nevertheless, there have been reports of considerable variability in response to IV hydralazine, potentially causing significant and undesirable drops in BP in acute scenarios [40]. Sodium nitroprusside relaxes the smooth muscle cells in both arteries and veins. It metabolizes to nitric oxide, which dilates venules and arterioles, reducing total peripheral resistance, making it effective for treating severe HTN. Its short half-life and rapid onset of action allow for precise BP titration. However, prolonged use (over 72 hours) or use in patients with compromised renal function can lead to complications such as hypotension and thiocyanate toxicity.

Diuretics

Diuretics lowers the BP by reducing extracellular and plasma volume. In infants, they are typically used for mild HTN caused by volume overload or as a second-line treatment when a single medication is insufficient to control BP. Additionally, diuretics can help improve lung function in infants with BPD. Thiazide diuretics, such as chlorothiazide and hydrochlorothiazide, are typically favored over loop diuretics (e.g., furosemide) when prescribing diuretics for the treatment of neonatal HTN. This preference is due to the higher likelihood of adverse effects associated with loop diuretics. Loop diuretics pose a greater risk of electrolyte imbalances, particularly hypokalemia, and can lead to hypercalciuria, potentially resulting in nephrocalcinosis.

Beta Blockers

Neonatal HTN is often treated with both oral and IV beta blockers. Nevertheless, caution should be exercised when administering these medications to infants with chronic lung disease due to the risk of bronchoconstriction. Propranolol has shown efficacy in managing neonatal HTN, with rare occurrences of side effects other than bradycardia. Labetalol exerts its effects on both alpha-1 and beta receptors, displaying a rapid onset of action and a duration lasting two to three hours. Typically, it is administered intravenously, either through a continuous infusion or intermittent doses. Labetalol may be particularly beneficial in managing HTN mediated by catecholamines and the central nervous system, as it does not lead to alterations in intracranial pressure, cerebral vasodilation, or tachycardia. Esmolol, on the other hand, is a beta-1 adrenergic blocker that is ultra-short-acting and cardioselective. Due to its quick onset of action (around 60 seconds) and relatively brief duration of action (10 to 20 minutes), it is a favorable option for treating severe symptomatic HTN.

Angiotensin-Converting Enzyme Inhibitor (ACEI) and Angiotensin Receptor Blocker (ARB) Classes

Given the importance of the RAAS for renal development, it is generally not recommended to give ACEI or ARB to neonates due to potential side effects. Ku [41] reported that adverse effects were observed in more than 20% of neonates administered an ACEI. The most prevalent complications included AKI, mortality hypotension, and hyperkalemia [42]. The significant disruption of the RAAS during fetal development can lead to oligohydramnios, calvarial defects, and the Potter sequence (which involves arthrogryposis, facial malformation, pulmonary hypoplasia, and death) [43]. Given that, with some degree of individual variation, typical glomerular maturation does not occur until 36 weeks of gestational age, it is generally advised to delay using RAAS blockers until 40-42 weeks postnatal age.

Acute severe HTN management in neonate

IV medicines are usually recommended in the event of acute and/or acute severe HTN. A continuous infusion of medicine is advised for neonates exhibiting signs of end-organ damage or hypertensive emergencies since it is simple to titrate dosage to meet target BP levels. Nicardipine or sodium nitroprusside are recommended drugs; however, due to the danger of thiocyanate poisoning, sodium nitroprusside must be used with caution. If an infant is to be handled with a continuous infusion, it is advised that arterial lines be inserted for continuous BP monitoring. If oscillometric cuffs are required, it is advised that BP be checked often (every five to fifteen minutes). Additional medications include IV labetalol or hydralazine dosed intermittently. When a newborn has a hypertensive emergency, it is advised to gradually lower their BP to prevent consequences including cerebral hemorrhage or cerebral ischemia. In general, it is advised to drop the BP by one third during the first six hours, then by another one third during the next 24 to 36 hours, and finally by the remaining amount during the next 48 to 72 hours [44]. Lowering the BP to less than the PMA of 95th percentile is the ultimate objective. Renovascular HTN caused by thromboembolism associated with umbilical catheter is usually temporary, with certain patients needing treatment for a short duration of weeks or months. HTN linked to AKI should subside as kidney function improves. HTN related to aortic coarctation generally resolves after successful correction, although these infants are susceptible to HTN recurrence in the future [44-45]. A retrospective investigation on infants with idiopathic HTN who were discharged from the NICU while receiving antihypertensive treatment was carried out by Xiao et al. [46]. They found that among this group of patients, CCBs were the most frequently prescribed class of antihypertensives (56%). After being discharged from the NICU, 60% of newborns continued to take antihypertensives, 26% did so after a year, and 7% did so after two years. Antihypertensive medication was not as likely to be required more than a year after discharge in cases when prenatal steroid treatment was administered.

## Conclusions

In conclusion, neonatal HTN is a multifaceted condition that demands meticulous attention from healthcare providers. From accurate BP measurement techniques to targeted treatment modalities and ongoing research endeavors, addressing neonatal HTN requires a holistic and collaborative approach. It is imperative to measure BP in newborn infants using a predetermined standard protocol that considers the influence of antenatal, perinatal, and postnatal factors that can impact neonatal BP. Additional significant variables that affect neonatal BP include postnatal age, gestational age, gender, and birth weight. Renovascular disease associated with umbilical artery catheterization is the most common underlying reason. Severe HTN, irrespective of the end-organ dysfunction, necessitates prompt management with IV medications and regular monitoring. As medical knowledge advances, the ability to diagnose, manage, and improve outcomes for neonates with HTN will undoubtedly continue to evolve, ultimately enhancing the quality of care provided to this vulnerable population.
